# Hydroxypropyl chitosan nail lacquer of ciclopirox-PLGA nanocapsules for augmented in vitro nail plate absorption and onychomycosis treatment

**DOI:** 10.1080/10717544.2022.2144543

**Published:** 2022-11-13

**Authors:** Eman Yahya Gaballah, Thanaa Mohammed Borg, Elham Abdelmonem Mohamed

**Affiliations:** Department of Pharmaceutics, Faculty of Pharmacy, Mansoura University, Mansoura, 35516, Egypt

**Keywords:** Ciclopirox, nanocapsules, hydroxypropyl chitosan, in vitro nail absorption, onychomycosis

## Abstract

Onychomycosis accounts for 90% of nail infections worldwide. Topical therapy provides localized effects with minimal adverse systemic actions, yet its effectiveness is limited by minimal drug permeation through the keratinized nail plate. Ciclopirox (CIX) is a FDA-approved broad-spectrum antimycotic agent. However, the complete cure with its nail lacquer (8% w/v) may continue for one year with a high cost. Therefore, poly lactide-co-glycolide (PLGA) nanocapsules (NCs) of CIX were prepared by nanoprecipitation and optimized through a 2^3^ factorial design to be incorporated into hydroxypropyl chitosan (HPCH) based nail lacquer. Nail hydration, *in vitro* nail absorption, minimum inhibitory concentration (MIC), inhibition zones and *ex vivo* fungal growth on nail fragments were evaluated. The optimized NCs of CIX based on 100 mg PLGA 2 A and lipoid S75 showed a mean diameter of 174.77 ± 7.90 nm, entrapment efficiency (EE%) of 90.57 ± 0.98%, zeta potential (ZP) of −52.27 ± 0.40 mV and a prolonged drug release. Nail lacquer of the optimized NCs exhibited a higher stability than NCs dispersion. Compared to CIX solution (1% w/v), the respective decrease in MIC for NCs and their lacquer was four- and eight-fold. The lacquer superiority was confirmed by the enhancement in the nail hydration and absorption by 4 and 2.60 times, respectively, relative to CIX solution and the minimal *ex vivo* fungal growth. Therefore, HPCH nail lacquer of (1% w/v) CIX-PLGA-NCs can be represented as a potential topical delivery system for enhanced in vitro nail absorption and therapeutic efficacy against onychomycosis at a low dose.

## 1. Introduction

Onychomycosis accounts for 90% of nail infections worldwide (Vlahovic, 2016). It is a chronic condition that often relapses after treatment (Marty et al., 2005). The main causative agent (about 60%) is *Trichophyton rubrum* (Flores et al., 2017). In comparison with the systemic therapy, topical treatment was recognized as an attractive alternative because it avoids the systemic side effects and drug interactions, as well as it allows a better patient compliance. However, the compact keratinized nail plate acts as a barrier against the efficient drug delivery using conventional topical formulations. The drawbacks of the currently employed penetration enhancing techniques hindered their applications (Dhamoon et al., 2019). Nail lacquers can provide an adequate drug concentration at the nail surface (Tabara et al., 2015). Water-insoluble monoester film-formers have no affinity to keratin in the nail plate (Sparavigna et al., 2008). Moreover, organic solvents frequently used to remove these insoluble films can damage the nail structure and increase the risk of reinfection (Piraccini et al., 2020). As well, some drugs may tend to crystalize after the rapid evaporation of the organic solvent which can hinder the drug diffusion through the nails (Flores et al., 2017). Therefore, nail lacquers based on water soluble film-former as hydroxypropyl chitosan (HPCH) that are easily removed with water were employed to improve the drugs adhesion and partition to the nails due to hydrogen bonding of free hydroxypropyl groups of HPCH with keratin (Sparavigna et al., 2014; Piraccini et al., 2020). Chitosan and its derivatives may exert antifungal activities possibly through interference with the fungal growth and the induction of proteinase inhibitors synthesis (Peng et al., 2005).

Greater potentiation in nail permeation has been recorded for nanostructures in comparison with free drugs (Flores et al., 2017). Polymeric nanocapsules (PNCs) are formed of a core enclosing a solid or a liquid bound by a polymeric shell to increase their stability (Dhamoon et al., 2019). The core is filled with a lipophilic solvent like oil to hold the hydrophobic drugs. Synthetic polymers which are highly stable like poly lactide-co-glycolide (PLGA) are usually used to prepare PNCs (Dhamoon et al., 2019). Encapsulating antifungals in PNCs provided an enhanced efficacy, a sustained release and an improved permeation (Soliman, 2017). Because of the low polymer content on the nanocapsular shell, much less inherent toxicity can be expected when compared to other nanoparticles as nanospheres (Aldalaen et al., 2019). The oil in PNCs can improve the drug diffusion through the microorganism membranes (Lboutounne et al., 2002). Also, the polymer adsorption at the interface due to hydrophobic surface properties as well as steric and electrostatic mechanisms can direct the drug to specific microorganism structures (Nhung et al., 2007). PNCs can deposit high drug amounts on the fungal surface due to their small size (Padmavathy & Vijayaraghavan, 2008).

Ciclopirox (CIX) is a FDA-approved broad-spectrum antimycotic that inhibits the metal-dependent enzymes in fungi that are responsible for the breakdown of toxic peroxides (Subissi et al., 2010). CIX nail lacquer (8%w/v) is widely used for nail fungal treatment (Subissi et al., 2010). However, the cost of treatment with CIX per patient cured (252 EURO) was greatly higher than other comparative antifungals commonly used for treatment of onychomycosis such as amorolfine (84 EURO) (Marty et al., 2005). Also, the complete cure may require treatment with CIX for one year or more (Piraccini et al., 2020). The lack of cost effectiveness and the long-term topical treatment may lead to the patient incompliance and the discontinuous treatment. The above-mentioned limitations of the topical therapy with CIX may necessitate a reduction in its dose and an improvement in its delivery and efficacy. This may require combining different strategies by utilization of a promising nanostructure as PNCs incorporated in a water-soluble nail lacquer to highly augment CIX efficacy. Therefore, the aim of this study was to prepare, characterize and optimize CIX-PNCs at a lower dose than that commonly used utilizing the biodegradable and biocompatible PLGA. This was followed by formulating the optimized PNCs into HPCH based nail lacquer to be assessed regarding the nail hydration and in vitro nail absorption of CIX in comparison with the drug solution (1%w/v). Also, the lacquer antifungal efficacy against *Trichophyton rubrum* was investigated through the evaluation of the effects on CIX minimum inhibitory concentration (MIC) and inhibition zone as well as via employing an *ex vivo* microbiological model.

## 2. Materials and methods

### 2.1. Materials

Ciclopirox (CIX) was purchased from 2A Biotech (Lisle, Illinois, USA). Acid terminated poly-lactide-co-glycolide (PLGA) polymers (50:50 grade 5002 A, molecular weight 17000 g/mol and 50:50 grade 5004 A, molecular weight 44000 g/mol) were kindly provided by Corbion (Gorinchem, Netherlands). Glyceryl monolinoleate (Maisine) was kindly provided by Gattefosse (Saint-Priest, France). Lipoid S75 was kindly provided by Lipoid AG (Schweiz, Switzerland). Span 60 was purchased from ITWCo. (Darmstadt, Germany). Tween 20 was obtained from Sigma-Aldrich (Saint Louis, MO, USA). Hydroxy-propyl chitosan (HPCH) was supplied by Xi’an Imaherb Biotech CO., Ltd (Xi’an Shanxi, China). Cetostearyl alcohol was obtained from Al-Gomhoria Co. (Cairo, Egypt). Acetone, methanol, ethanol, and acetonitrile were purchased from Fisher Scientific (Leicestershire, UK). Amicon® Ultra-4 centrifugal filter units (4 mL, 10 KDa cutoff units), were purchased from Merck CO. (California, USA). Spectrapor® membrane, MW cutoff: 12,000-14,000 Da, was purchased from Spectrum Medical Industries Inc. (Los Angeles, USA). Sabouraud’s dextrose agar was purchased from Oxoid Ltd (Basingstoke, UK). *Trichophyton rubrum* strains were obtained from Assiut University Moubasher Mycological Center (Assiut, Egypt). All other chemicals were of fine analytical grade.

### 2.2. Experiment design and optimization

To determine the optimized formula to be further evaluated, screening of CIX-PLGA-NCs formulations using a two-level and three-variable strategy with 2^3^ full factorial design was done using Design-Expert V.13 software (Stat-Ease, Minneapolis, USA). Polymer type (PLGA 2 A and PLGA 4 A) represented level 1 and level 2 of X_A_, respectively. Polymer amounts of 75 mg and 100 mg represented minimum and maximum levels of X_B_, respectively. Level 1 and level 2 of X_C_ were represented by the type of the organic phase stabilizer (Lipoid S75 and Span 60, respectively). These were selected as three critical independent variables, thus eight formulations (F1–F8) were prepared and evaluated (Table 1). For each formula, dependent variables including particle size (PS), polydispersity index (PDI), zeta potential (ZP) and percent entrapment efficiency (EE%) were evaluated. The complete first order polynomial regression equation was generated as follows (Nair et al., 2011):

$$Y=\;\beta 0+\;\beta {1\;X}_{A}+\;\beta {2\;X}_{B}+\;\beta {3\;X}_{C}+\;\beta {4\;X}_{\text{AB}}+\;\beta {5\;X}_{\text{AC}}+\;\beta {6\;X}_{\text{BC}}+\;\beta {7\;X}_{\text{ABC}}$$

**Table 1.** The independent formulation’s variables and their levels of 2^3^ full factorial design, (B) CIX-PLGA-NCs formulations codes and the resultant dependent variables.

(A)							
Independent variable			Name		Type	Levels	
						Minimum level	Maximum level
X_A_			Polymer type		Categoric	PLGA 2 A	PLGA 4 A
X_B_			Polymer amount		Numeric	75 mg	100 mg
X_C_			Organic phase stabilizer type		Categoric	Lipoid S75	Span 60
**(B)**							
	Independent variables (X) (Factors)			Dependent variables (Y) (Responses)			
Formula	X_A_	X_B_	X_C_	Y1	Y2	Y3	Y4
F1	2A	75	Lipoid S75	69.33 ± 1.16	208.13 ± 1.86	0.32 ± 0.01	−42.97 ± 1.11
F2	4A	75	Lipoid S75	71.67 ± 1.53	248.73 ± 6.90	0.35 ± 0.03	−40.63 ± 1.03
F3	2A	100	Lipoid S75	90.57 ± 0.98	174.77 ± 7.90	0.33 ± 0.05	−52.27 ± 0.40
F4	4A	100	Lipoid S75	88.67 ± 1.53	242.0 ± 12.69	0.35 ± 0.05	−50.53 ± 0.61
F5	2A	75	Span 60	74.67 ± 0.58	247.9 ± 7.27	0.40 ± 0.01	−33.97 ± 3.64
F6	4A	75	Span 60	79.33 ± 4.73	290.6 ± 11.18	0.44 ± 0.05	−41.63 ± 0.12
F7	2A	100	Span 60	89.00 ± 1.00	213.53 ± 0.95	0.35 ± 0.01	−41.27 ± 1.68
F8	4A	100	Span 60	90.40 ± 0.69	271.97 ± 4.25	0.49 ± 0.04	−43.40 ± 0.96

* Data are represented as mean ± S.D (*n* = 3). Y1: entrapment efficiency% (EE%), Y2: particle size (PS, nm), Y3: polydispersity index (PDI), Y4: zeta potential (ZP, mV).

Where;

**Table:** 

Y	is the dependent variable
β0	is the arithmetic mean response of the eight runs
β1, β2 and β3	linear coefficients
β4, β5 and β6	interaction coefficients between the two independent variables
β7	interaction coefficients between the three independent variables

### 2.3. Preparation of CIX loaded PLGA NCs

CIX-PLGA-NCs were prepared employing nanoprecipitation method due to its simplicity, low cost, high encapsulation efficiency and reproducible size of the produced NCs (Mora-Huertas et al., 2010; Klippstein et al., 2015). Briefly, 5 mL acetone were added to dissolve 10 mg CIX, 0.50 mL maisine, 75 mg or 100 mg PLGA (2 A or 4 A) and 50 mg (1% w/v) lipoid S75 or span 60 as organic phase stabilizers. The organic phase was injected dropwise into a 10 mL aqueous phase stabilized by 100 mg tween 20 (1% v/v) with stirring (MS300HS, MTOPS Corp., Korea) at room temperature till complete evaporation of acetone and to obtain a final volume of 10 mL and a drug concentration of 1 mg/mL. NCs dispersions were kept in the refrigerator (4-8 °C) for further analysis (Aldalaen et al., 2019).

### 2.4. Preparation of nail lacquer of the optimized formula

For further evaluation studies, the optimized CIX-PLGA-NCs with a drug concentration of 1 mg/mL were incorporated into HPCH based nail lacquer (Mailland, 2014). Briefly, 0.10 g cetostearyl alcohol was solubilized in 1 mL ethanol and then 10 mL of the optimized NCs dispersion were added under the magnetic stirring (MS300HS, MTOPS Corp., Korea). After homogenization, 0.10 g of HPCH was added under the magnetic stirring for 1 h till complete consistency. The lacquer was stored in the refrigerator (4-8 °C) for further analysis (Mailland, 2014, Flores et al., 2017).

### 2.5. Characterization of CIX-PLGA-NCs

The effects of the selected independent variables on the evaluated dependent variables including PS, PDI, ZP and EE% were investigated and expressed as response three-dimensional surface plots in which X_B_ factor was fixed at its high and low levels, while X_A_ and X_C_ were varied over the range set. pH and viscosity of the optimized NCs and their lacquer were measured.

#### 2.5.1. Determination of total drug content and entrapment efficiency (EE%)

Total drug content either free or encapsulated was measured by a complete dissolution of NCs dispersion in acetonitrile as a common solvent for PLGA and CIX (Granata et al., 2022). To separate the free drug, NCs dispersion was placed in Amicon® Ultra 4 centrifugal filter units (4 mL −10 KDa cutoff unit) and centrifuged (CE16-4X100RD, ACCULAB, NY, USA) at 6000 rpm for 30 min at 25 °C. The ultrafiltrate was properly diluted with methanol and analyzed spectrophotometrically at 248 nm (ultraviolet/visible [UV/VIS] spectrophotometer; JASCO, Tokyo, Japan) against a blank of plain NCs treated the same. EE % was calculated as follows: (Aldalaen et al., 2019).

$$\text{EE}\%=\frac{\left(\text{Total}\;\text{drug}-\text{Free}\;\text{drug}\right)}{(\text{Total}\;\text{drug})}\times 100$$

#### 2.5.2. Ps, PDI and ZP determination

The mean PS, PDI and ZP of the prepared CIX-PLGA-NCs were determined (Malvern Instruments Ltd, Malvern, Worcestershire, UK). The samples were diluted with double deionized water (1:500) before analysis (Flores et al., 2017).

#### 2.5.3. Morphological analysis

The nanocapsular dispersion was diluted ten-fold with double deionized water and sonicated for 2 min (MS300HS, MTOPS Corp., Korea). One drop of the diluted sample was dropped onto carbon-coated copper grid and the excess liquid was removed with a filter paper. After complete drying at room temperature, the image capture (TEM-2100, JEO, Tokyo, Japan) at an accelerating voltage of 160 kV and analysis were done using Digital Micrograph and Soft Imaging Viewer software .

#### 2.5.4. pH and relative viscosity measurements

pH of the optimized NCs dispersion and its lacquer was measured using a pre-calibrated digital pH-meter (Beckman Instrument Fullerton, Germany). Ostwald viscometer was used to determine the flow time at 25 °C for double deionized water and compare it to that of the evaluated formulation (Meenakshi and Sudhan, 2015).

### 2.6. In vitro release studies and kinetic analysis

*In vitro* release of CIX from the optimized NCs and their nail lacquer was done in a two-compartment dialysis system using a cellulose membrane (Spectrapor® membrane, MW cutoff: 12,000-14,000 Da) of 2.50 cm diameter (Moraes et al., 2009). One hundred milliliters of phosphate buffer solutions at pH 5.8 (specific pH for nails) (Thatai & Sapra, 2018) and pH 7.4 (Flores et al., 2017) were used as release media. To ensure sink condition, 1%v/v tween 20 was added to the release media (Łukasiewicz et al., 2016). The drug dispersion in double deionized water and its aqueous solution containing 1%v/v tween 20 were used as controls. Two milliliters of the tested samples were placed in the donor compartment. The release media were maintained at 32 ± 2 °C which is reported to be the temperature of the nail surface (Elsherif et al., 2017) and stirred at 100 rpm. One milliliter samples were withdrawn at 1, 2, 3, 4, 5, 6, 7, 8 and 24 h to be analyzed spectrophotometrically (ultraviolet/visible [UV/VIS] spectrophotometer; JASCO, Tokyo, Japan) at 251 nm for both media against the respective blank treated the same. The experiments were performed in triplicate and the cumulative percentage CIX released was calculated. First-order and zero-order (Martin et al., 1993) as well as diffusion (Higuchi, 1963) and Korsmayer–Peppas kinetic (Ritger and Peppas, 1987) models were used to explain *in vitro* release mechanism.

### 2.7. Storage stability study

The optimized NCs and their lacquer were packed in amber glass vials and maintained at refrigerated (5 ± 1 °C) and room (25 ± 2 °C/60 ± 5% relative humidity) temperatures for three months. Physical appearance, pH, relative viscosity, PS, PDI, ZP, drug content and EE% were evaluated at the beginning of the study and monthly (Klippstein et al., 2015).

### 2.8. Nail hydration and in vitro nail absorption

#### 2.8.1. Preparation of nail clippings

Nail clippings of the index, middle and ring fingers were collected from healthy human volunteers (20–40 years) using nail clippers (Bseiso et al., 2016). They were washed with deionized water, wiped with tissue paper, dried at 37 °C for 24 h and stored in air tight containers till use (Bseiso et al., 2016).

#### 2.8.2. Nail hydration procedure

The reported antifungal effects of (≥ 70% v/v) ethanol (Loewenthal, 1961) have been verified by the results of preliminary studies (data not shown) that revealed ethanol interference with the fungal growth, and hence with the assessement of inhibition zone and *ex vivo* antifungal efficacy. Such interference was indicated by the complete growth inhibition of *Trichophyton rubrum* on agar blocks and nail fragments in contrast to 1% v/v tween 20 solution of the free drug that showed a moderate fungal growth as discussed later. As well, a preliminary study to determine inhibition zone diameter using 70% v/v ethanolic solution of the free drug suggested ethanol diffusion through the agar inhibiting the fungal growth in presence of other studied samples including saline. The agar plate was well-covered, so ethanol evaporation was counteracted. Therefore, HPCH nail lacquer of free CIX and the marketed products have not been used for comparison due to their high ethanol content (96% v/v) to solubilize CIX that would interfer with the fungal growth. Consequently, these preparations have not been used in nail hydration and *in vitro* nail plate absorption. Tween 20 (1% v/v) aqueous solution of the free CIX was selected as a control because tween 20 completely solubilized the drug and it was the dispersing aqueous phase of the prepared NCs. As well, it did not interfere with the above-mentioned studies. Three groups were designated for this experiment. For comparison, aqueous CIX (1%w/v) solution containing 1%v/v tween 20 was used. The nail clippings were immersed in 1 mL of either double deionized water as a control (group 1), the nail lacquer of the optimized NCs (group 2) or the drug solution (group 3). Fifty milligrams of the washed and dried nail clippings were placed in tightly closed glass vials to be stored at room temperature for 24 h. The nail clippings were wiped with tissue paper to remove the surface fluids and reweighed to calculate the weight gain and the hydration enhancement factor after 24 h (HE_24_): (Chouhan & Saini, 2012, Bseiso et al., 2016)

$${\text{HE}}_{24}=\frac{\text{Weight}\;\text{gain}\;\text{of}\;\text{nail}\;\text{clippings}\;\text{of}\;\text{groups}\;2\;\text{or}\;3}{\text{Weight}\;\text{gain}\;\text{of}\;\text{nail}\;\text{clippings}\;\text{of}\;\text{control}\;\text{group}}$$

#### 2.8.3. In vitro nail plate absorption and desorption

To quantify the absorbed and desorped CIX, the nail clippings of groups 2 and 3 were washed with deionized water then methanol to remove any drug traces on the surface. They were dissolved in 1 mL of 1 M sodium hydroxide by stirring (MS300HS, MTOPS Corp., Korea) overnight and then filtered through 0.22 µm syringe filter. The filtrate was properly diluted with methanol and CIX accumulated in the nail clippings was determined spectrophotometrically at 248 nm (ultraviolet/visible [UV/VIS] spectrophotometer; JASCO, Tokyo, Japan) against the respective blank treated the same. The enhancement factor (EF_nail_) indicating the improvement of CIX penetration into the nail clippings from HPCH lacquer compared to the drug solution was calculated according to the following equation (Palliyil et al., 2013):

$${\text{EF}}_{\text{nail}}=\frac{\text{Extracted}\;\text{drug}\;\text{percentage}\;\text{in}\;\text{nail}\;\text{clippings}\;\text{of}\;\text{group}\;2\;}{\text{Extracted}\;\text{drug}\;\text{percentage}\;\text{in}\;\text{nail}\;\text{clippings}\;\text{of}\;\text{group}\;3}$$

### 2.9. Assessment of microbiological efficacy

#### 2.9.1. Inoculum preparation

A stock inoculum suspension of *Trichophyton rubrum* was prepared using 14-days old cultures grown on Sabouraud’s dextrose agar (SDA) slants at 28 °C. The fungal colonies were covered with 2 mL of Sabouraud’s dextrose broth (SDB) ­followed by a gentle scratching of the agar’s surface with a sterile swab, resulting in a suspension of conidial and hyphal fragments that was transferred to a sterile tube and kept for 5 to 10 minutes at room temperature for sedimentation of heavy particles keeping micro-conidia suspended. Filtration was performed to retain hyphal fragments but permit the passage of dermatophyte micro-conidia to be used later (Motedayen et al., 2018). The turbidity of the fungal suspension was measured spectrophotometrically (ultraviolet/visible [UV/VIS] spectrophotometer; JASCO, Tokyo, Japan) at 600 nm and adjusted with SDB to match the 0.5 McFarland standard which is equivalent to 10^5^-10^6^ CFU/mL (Marcato et al., 2012).

#### 2.9.2. Minimum inhibitory concentration determination

MIC was determined based on Broth Microdilution method according to Clinical and Laboratory Standards Institute (CLSI M38) using 96-flat-bottomed well microplates. Serial two-fold dilutions of drug were prepared from a stock solution in DMSO. SDB was used to dilute the optimized NCs and the nail lacquer. CIX concentrations range was 0.03-32 µg/mL (Wayne, 2008). Briefly, 100 µL of each concentration were inoculated to the wells. Then, 100 µL of inoculum suspension in SDB (1x10^3^ - 3x10^3^ CFU/mL) were added to each drug well. Growth, solvent and sterility controls as well as blanks of both the optimized NCs dispersion and its nail lacquer were also investigated. Plates were incubated at 28 °C for 4 days. Results were evaluated visually (Motedayen et al., 2018).

#### 2.9.3. Inhibition zone determination

The selected NCs lacquer containing 1%w/v CIX, its blank and aqueous drug (1%w/v) solution containing 1%v/v tween 20 as a positive control were evaluated for their antifungal activity against *Trichophyton rubrum* applying the agar diffusion technique (Bseiso et al., 2016). Saline was included as a negative control. Sabouraud’s dextrose agar growth medium fortified with levofloxacin to guard against bacterial growth was added to a sterile petri dish (El-Emam et al., 2020). After agar solidification, 1 mL of the fungal suspension (10^5^ CFU/mL) was added and the agar plate was moved roundly in a clockwise and anticlockwise directions. Four wells of 6 mm internal diameter were made in the agar using a sterile cork-borer. Each well was filled with 0.10 mL of the above-mentioned tested samples. The plates were then incubated at 28° C for three days to enhance the fungal growth (Bseiso et al., 2016). The antifungal activity was assessed by measuring the diameter (mm) of the formed inhibition zones surrounding the formulations using a scale.

#### 2.9.4. Assessment of antifungal efficacy against onychomycosis

The therapeutic efficacy of the optimized NCs nail lacquer was evaluated using a previously adopted *ex vivo* onychomycosis model (Flores et al., 2017). Healthy female volunteers with no history of receiving any antifungal treatment in the last six months kindly donated the nails clippings (*n* = 9). Nail fragments were sterilized in autoclave before use. For comparison, CIX (1%w/v) aqueous solution containing 1%v/v tween 20 as a positive control was used. The blank lacquer and saline as a negative control were also used. The fungal dispersion of *Trichophyton rubrum* (10^5^ CFU/mL) was inoculated into SDA medium containing levofloxacin prior to solidification and shaked for uniform fungal distribution to be then poured into sterile petri dishes to be cut into blocks (about 3 mm^2^) after solidification. The ventral parts of the nail fragments were placed on the inoculated agar blocks while the dorsal parts were exposed. The blocks were transferred to a small sterile petri dish of a diameter equal to 5 cm. Air humidity was maintained by using a larger petri dish (9 cm) containing 4 mL of the sterile water. The agar blocks were daily hydrated with a sterile SDB. CIX tested formulations were applied once a day for 6 days on the dorsal parts by a sterile swab to completely cover the fragments surface. The petri dishes were maintained at 28 °C in the incubator and at the end of the 6 days the fungal growth on the nails was recorded visually.

### 2.10. Statistical analysis

Statistical analysis of the data of stability, nail hydration, and inhibition zone diameter was accomplished through (ANOVA) one-way analysis of variance, followed by Tukey-Kramer multiple comparisons test. The data of pH and viscosity as well as *in vitro* nail absorption and desorption were statistically analyzed applying Student’s *t*-test (unpaired). GraphPad Prism version 5.00 (GraphPad software, San Diego, CA, USA) was employed at a significance level of *p* < 0.05 to perform the statistical analysis. All data were interpreted as the mean values ± standard deviation (SD), *n* = 3. The experimental 2^3^ full factorial design was evaluated based on ANOVA employing Design-Expert V.13 software (Stat-Ease, Minneapolis, USA). Statistically significant F-value (*p* < 0.05) and adjusted determination coefficients (adjusted R^2^) between 0.80-1 were the criteria for the chosen model validation (Aman et al., 2018).

## 3. Results and discussion

### 3.1. Characterization and optimization of CIX-PLGA-NCs

#### 3.1.1. Effects of formulation independent variables on EE%

As represented in Table 1, all CIX-PLGA-NCs formulations possessed EE% ranged from 69.33 ± 1.16% to 90.57 ± 0.98%. The obtained polynomial equation represents the linear regression model for EE% is as follows:

EE% = +81.7 +0.8125 A +7.95 B +1.65 C −0.9375 AB +0.7042AC −1.6 BC +0.1208 ABC

Where: F = 62.25, *ρ* < 0.0001, and adjusted R^2^=0.9491.

**Figure 1.**
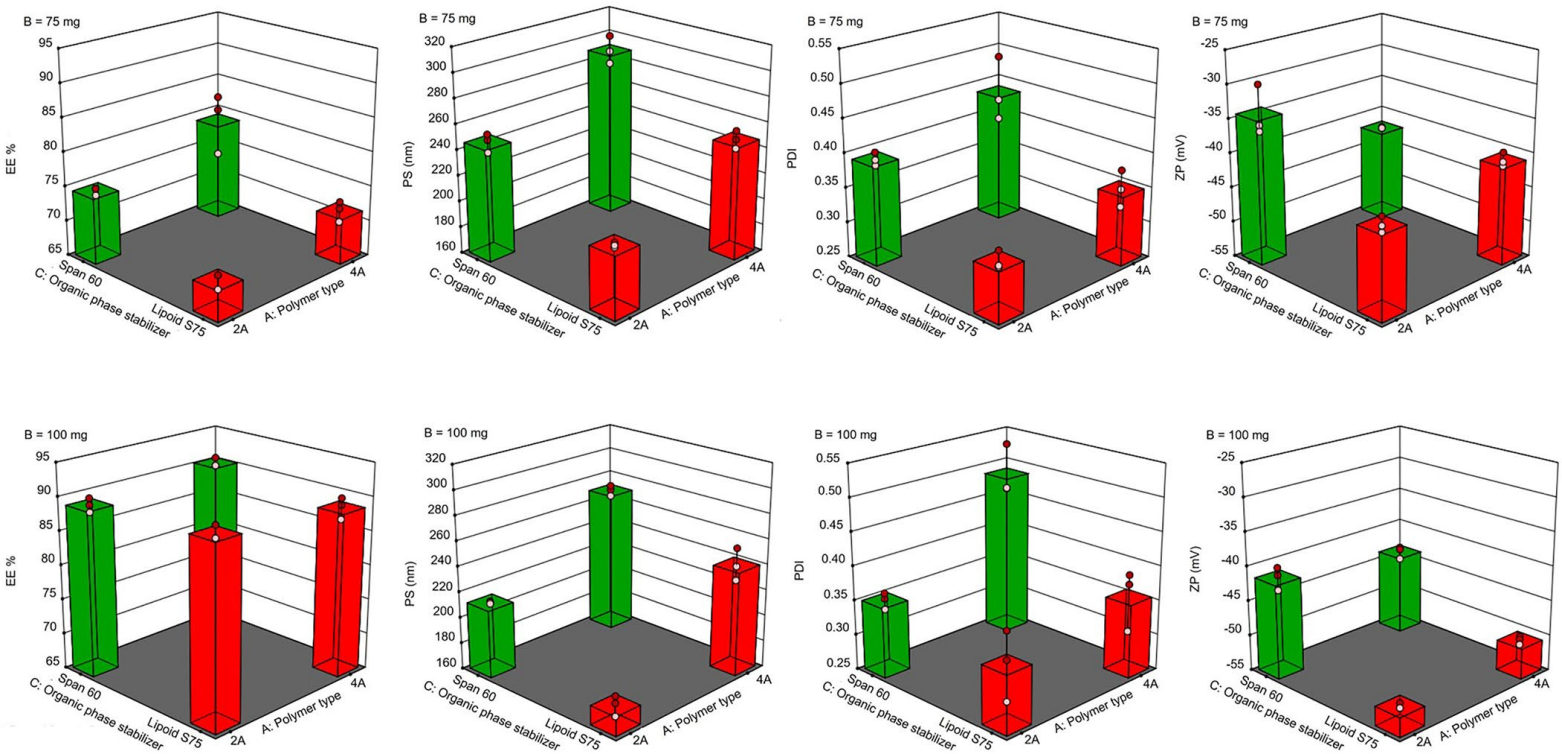
Three- dimensional surface graphs showing the effects of interaction between the type of both polymer (X_A_) and organic phase stabilizer (X_C_) on %EE, PS, PDI and ZP at the minimum and the maximum level of polymer amount (X_B_), respectively.

The above equation represents the quantitative effects of formulation independent variables which are polymer type (X_A_), polymer amount (X_B_) and type of organic phase stabilizer (X_C_), on EE%. The increase in EE% was observed with the use of maximum level of X_A_ (4 A) particularly when the value of (X_C_) is also maximized by utilization of span 60 as shown by the positive interaction coefficient between X_A_ and X_C_ (+0.7042 AC). Concerning the polymer amount (X_B_), the maximum level (100 mg) was shown to increase EE% greatly when compared to the minimum level (75 mg) as indicated by the highest coefficient value (+7.95 B) that suggested that the greatest positive effect on EE% was caused by the polymer amount (X_B_). This may explain that the maximization of polymer amount of either type to 100 mg predominated the effects of other variables as polymer type (X_A_) and type of organic phase stabilizer (X_C_). In accordance, NCs containing 100 mg of either polymer with each of lipoid S75 (F3 and F4) or span 60 (F7 and F8) provided EE% ≥ 88.67 ± 1.53%. As well, the effect of the type of organic phase stabilizer (X_C_) was abolished when the polymer amount (X_B_) was maximized to 100 mg. Hence, the effects of other dependent variables were assessed to determine the optimized formula to be further investigated. The results illustrated in Table 1 were in agreement with those represented in Figure 1.

The greater polymer amount as well as the use of 4 A type with high molecular weight (44000 g/mol) could result in more viscous organic phase which in turn would hinder the drug diffusion to the aqueous phase (Abd El Hady et al., 2019). Also, the rise in the polymer amount may result in a faster polymer precipitation in the aqueous phase, allowing less time for the drug to diffuse out of NCs (Abd El Hady et al., 2019). Surfactants with longer alkyl chains as span 60 generally produce larger particles allowing a greater drug entrapment (Hao et al., 2002).

#### 3.1.2. Effects of formulation independent variables on PS and PDI

According to Table 1, formulations showed PS at the range of 174.77 ± 7.90 nm to 290.60 ± 11.18 nm with narrow PS distribution (PDI < 0.50) indicating homogenous NCs dispersions. The obtained polynomial equations for PS and PDI are as follows:

PS = +237.2 +26.12 A −11.64 B +18.80 C +5.3 AB −0.8375 AC −1.61 BC −1.36 ABC

Where: F = 70.08, ρ < 0.0001, and adjusted R^2^=0.9546.

PDI = +0.3804 +0.0292 A +0.0033 B +0.0388 C +0.0108 AB +0.0158 AC −0.0013 BC +0.0120 ABC

Where: F = 8.34, ρ = 0.0002, and adjusted R^2^=0.6908.

Great effects of polymer type (X_A_) and organic phase stabilizer (X_C_) were suggested by the respective relatively high linear coefficients of +26.12 and +18.80. Positive signs indicated that the increase in PS due to the maximization of the independent variable levels of X_A_ (4 A type) and X_C_ (span 60). This may be due to the higher molecular weight of 4 A grade (44000 g/mol) when compared to 2 A type of (17000 g/mol). Similarly, span 60 as a high level of X_C_, obviously (F5-F8) produced larger NCs when compared to those based on lipoid S75 (F1-F4) possibly due to the shorter alkyl chain and the higher HLB value of lipoid S75 (7-9) than span 60 (4.70) resulting in colloidal dispersions with smaller particle size in case of the former (Motawea et al., 2018). On the other hand, the negative sign in the linear coefficient of X_B_ (-11.64) may indicate that the high amount of either polymer type provided smaller NCs. Higher polymer amounts may result in an increased viscosity of the organic phase which might counteract the particles diffusion and Ostwald ripening, so smaller NCs were obtained (Abd El Hady et al., 2019). Therefore, it can be concluded that to obtain a desired small PS, both X_A_ and X_C_ levels should be minimized to 2 A type and lipoid S75, respectively, while X_B_ level must be maximized to 100 mg. In agreement, F3 prepared utilizing 2 A PLGA (100 mg) and lipoid S75 exhibited the smallest PS (174.77 ± 7.90 nm). As noticed in PDI polynomial equation, a relatively small positive linear coefficient value was estimated for all the studied variables. This may indicate that the individual effect of each variable on PDI value remains insignificant.

#### 3.1.3. Effect of formulation independent variables on ZP

NCs stability against aggregation was suggested by the negative ZP in the range of −33.97 ± 3.64 mV to −52.27 ± 0.40 mV possibly because of the negatively charged acid terminated PLGA which formed the external layer of NCs (Mora-Huertas et al., 2010). Keratin is negatively charged at pH above 5, and hence an increased *in vitro* nail absorption of the negatively charged NCs through the nail plate can be expected due to the high repulsion forces with the nail keratin fibers (Elsherif et al., 2017). The polynomial equation for ZP is as follows:

ZP = −43.33 −0.7167 A −3.53 B +3.27 C +0.6167 AB −1.73 AC +1.27 BC +0.7667 ABC

Where: F = 40.51, ρ < 0.0001, and adjusted R^2^=0.9232.

**Figure 2.**
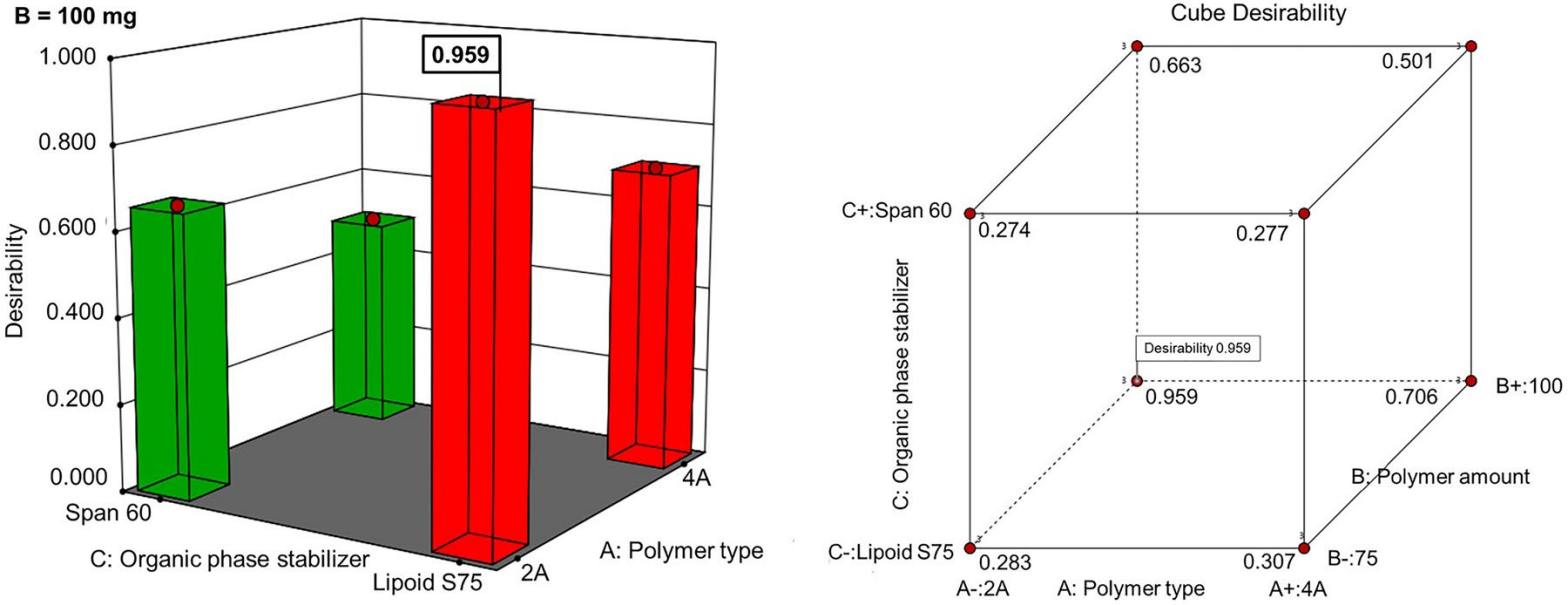
Three- dimensional surface and cube graphs showing the desirability values of the suggested optimization solutions by 2^3^ full factorial design.

The negative sign of the coefficient for X_B_ (-3.53) may indicate that the high level of such variable (100 mg polymer) can result in lower, but more negative ZP values that are required for more effective repulsion with keratin fibers and hence more potentiated nail plate absorption. This effect might be minimal in case of the polymer type as suggested by much lower numerical value of the coefficient for X_A_. These results can be explained on the basis that the greater polymer amount could provide more carboxylic groups resulting in higher negative surface charge of the prepared NCs. The relatively high numerical values and the positive sign of the coefficient for X_C_ (+3.27) was observed suggesting that the low level of such variable (lipoid S75) can result in lower more negative ZP values possibly because of the presence of fatty acids in this stabilizer (de Araújo et al., 2007). In agreement, the highest ZP was attained on use of 100 mg PLGA A2 and Lipoid S75 in NCs preparation (F3) (Table 1 and Figure 1). The results collectively verified that NCs formulaion containing 100 mg PLGA 2 A and Lipoid S75 (F3) has the highest EE%, the smallest PS, PDI value < 0.50 and a relatively high negative ZP. In agreement, the optimized formula suggested by Design-Expert V.13 software was F3 that showed the highest desirability coefficient equal to 0.959 (Figure 2). Therefore, this formula was chosen to be incorporated into HPCH nail lacquer and further investigated.

#### 3.1.4. Morphological analysis of the optimized NCs

**Figure 3.**
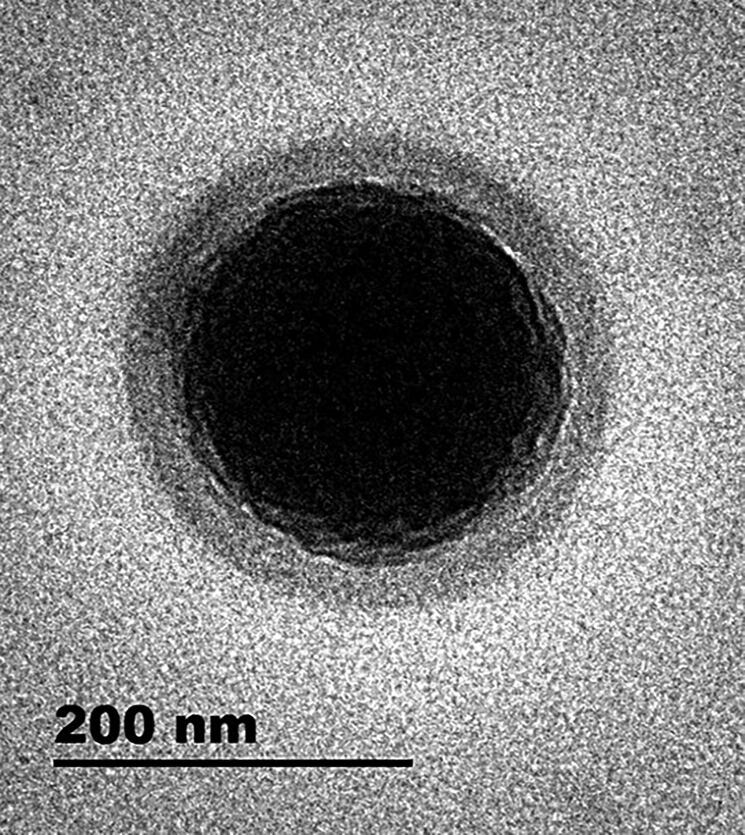
TEM micrograph of the optimized NCs (F3).

TEM images exhibited spherical NCs with a core-coat structure (Figure 3). PLGA shell of NCs has been represented as a predominant protectant against the rapid release of the drug incorporated in the oily core (Mora-Huertas et al., 2010). In agreement with PS measurements, NCs appeared smaller than 200 nm. Nanostructures as NCs have been found to potentiate the nail permeation (Flores et al., 2017).

#### 3.1.5. pH and relative viscosity measurements

The optimized NCs (F3) and its HPCH nail lacquer (L3) showed average low pH of 3.46 ± 0.01 and 3.97 ± 0.15, respectively, possibly due to the ionization of acidic groups of PLGA polymer chain (de Melo et al., 2011). The basic nature of HPCH may account for the significantly (*p* < 0.05) higher average pH values of the lacquer when compared to that of the optimized NCs dispersion (Bansal et al, 2011). The average relative viscosity of the optimized NCs was 1.2 ± 0.01 CP. The significantly (*p* < 0.05) higher viscosity of its nail lacquer (4.23 ± 0.06 CP) relative to that of NCs dispersion is expected to prolong the contact time on the nail plate that could permit a proper hydration and a drug absorption across the nails as well as an improved storage stability due to the hindered aggregation. The higher viscosity of the lacquer when compared to that of dispersion can be explained by the effects of HPCH as a thickening agent.

### 3.2. In vitro release studies and kinetic analysis

**Figure 4.**
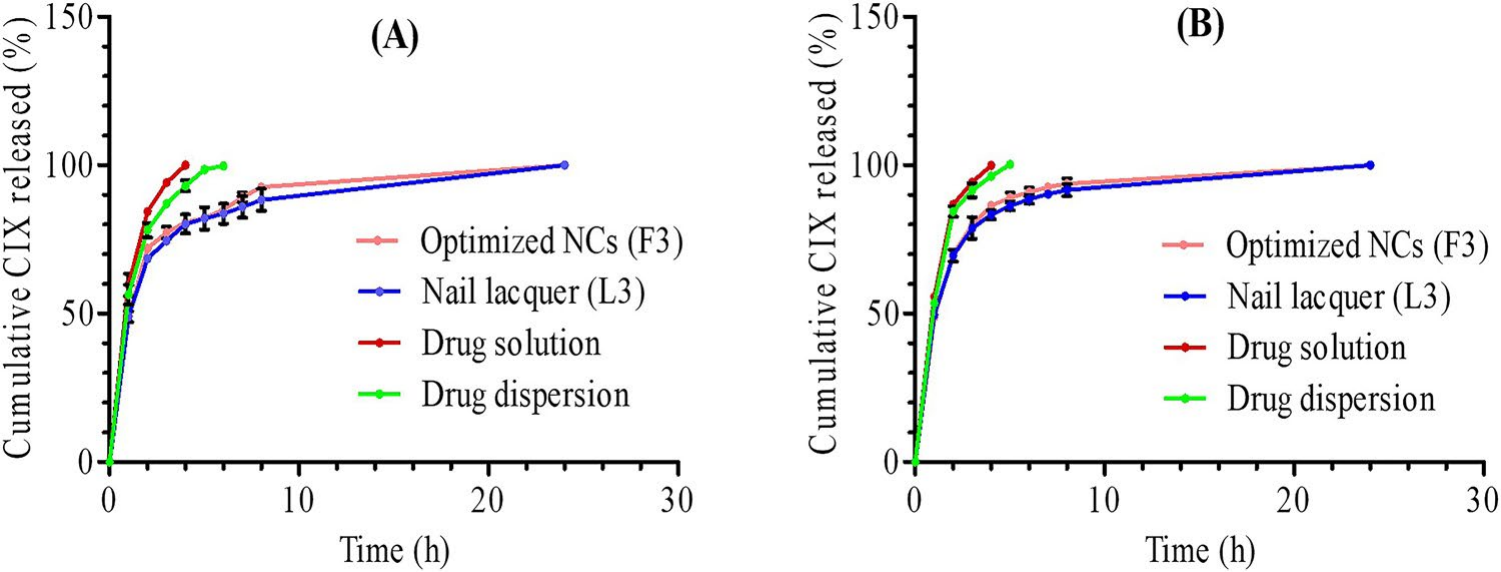
*In vitro* release profiles of CIX optimized NCs (F3) and their nail lacquer compared to free CIX solution and dispersion at (A) pH 5.8 and (B) pH 7.4.

*In vitro* release profiles of CIX from the optimized NCs (F3) and its HPCH nail lacquer in phosphate buffers of pHs 5.8 and 7.4 in comparison with the free drug in both solution and dispersion forms are illustrated in Figure 4(A and B, respectively). At both media, a rapid release during the first 4 hours was followed by a sustained release till 24 h. Similar results were reported for PLGA-NCs (Maribel et al., 2019). The initial fast release could be attributed to the desorption of the drug on NCs surface. The sustained release phase may be owing to the drug encapsulated in the oil core that was protected by PLGA coat as indicated by TEM examination (Flores et al., 2017). Therapeutic drug concentrations can be attained due to the rapid drug release, while the sustained drug release could maintain these concentrations (Mohamed et al., 2018). As well, the drug gradual release may lead to the reduction in the application frequency enhancing the patient compliance. *In vitro* release of CIX from both formulations during the rapid and sustained release phases can be explained by first order model (Table 2(A and B)). Fickian diffusion mechanism (*n* < 0.45) in which the release is assumed to occur by the molecular diffusion of the drug due to a chemical potential gradient, was found to describe the drug release during the two phases from F3 and its lacquer at the two media (Singhvi & Singh, 2011).

**Table 2.** Kinetic analysis of CIX release data at (A) pH 5.8 and (B) pH 7.4.

(A)							
Formula code	Correlation coefficient (r^2^)			Release order	Korsmeyer Peppas		Main transport mechanism
	Zero order	First order	Higuchi model		n	r^2^	
(F3)							
Rapid release phase	0.885	0.996	0.995	First	0.168	0.999	Fickian
Sustained release phase	0.723	0.978	0.864	First	0.103	0.882	Fickian
(L3)							
Rapid release phase	0.915	0.997	0.999	Higuchi	0.226	0.996	Fickian
Sustained release phase	0.741	0.999	0.983	First	0.124	0.990	Fickian
**(B)**							
Formula code	Correlation coefficient (r^2^)			Release order	Korsmeyer Peppas		Main transport mechanism
	Zero order	First order	Higuchi model		n	r^2^	
(F3)							
Rapid release phase	0.958	1	0.995	First	0.306	0.998	Fickian
Sustained release phase	0.58	0.997	0.974	First	0.065	0.985	Fickian
(L3)							
Rapid release phase	0.952	0.994	0.988	First	0.264	0.993	Fickian
Sustained release phase	0.597	0.999	0.985	First	0.085	0.993	Fickian

n:diffusional exponent, F3: the optimized nanocapsules (NCs), L3: hydroxypropyl chitosan (HPCH) nail lacquer of the optimized NCs.

### 3.3. Storage stability study

At refrigerator temperature, there were insignificant changes in the majority of the average examined parameters of the optimized NCs (F3) over the three months when compared to those initially determined (Table 3A). These results can be attributed to the relatively high negative ZP that is expected to hinder NCs aggregation. As well, tween 20 as a hydrophilic nonionic surfactant was used in the aqueous phase to coat and stabilize the outer surface of NCs (Aldalaen et al., 2019). However, there was a significant (*p* < 0.05) decrease in EE% during the second and third months and in ZP during the third month relative to the initial values possibly due to PLGA hydrolysis to some extent due to the acidic conditions (Mora-Huertas et al., 2010). In contrary, the nail lacquer exhibited insignificant changes regarding the examined parameters when compared to those initially determined (Table 3A) probably due to the signifcantly (*p* < 0.05) higher viscosity of the lacquer (4.23 ± 0.06 CP) relative to the selected NCs dispersion (1.2 ± 0.01 CP) that possibly hindered NCs aggregation. In addition, the insignificantly changed ZP is expected to largely hinder NCs aggregation. The lower negative zeta potential of the lacquer relative to the optimized NCs can be attributed to the positively charged HPCH.

**Table 3.** Storage stability of the optimized NCs dispersion and its nail lacquer at (A) refrigerator temperature (5 ± 1 °C) and (B) ambient conditions (25 ± 2 °C/60 ± 5% RH; relative humidity).

**(A)** Refrigerator temperature (5 ± 1 °C)							
Time	% Drug Content	% EE	PS (nm)	PDI	ZP (mV)	pH	Viscosity (CP)
(F3)							
0	100.20 ± 0.18	90.57 ± 0.98	174.70 ± 7.91	0.33 ± 0.05	−52.30 ± 0.40	3.46 ± 0.01	1.20 ± 0.01
1	99.70 ± 0.58	90.14 ± 0.24	176.30 ± 2.11	0.34 ± 0.01	−51.80 ± 0.10	3.42 ± 0.01	1.23 ± 0.02
2	99.67 ± 0.70	88.88 ± 0.44^#^	171.40 ± 3.58	0.45 ± 0.01	−51.30 ± 0.70	3.40 ± 0.01	1.21 ± 0.01
3	99.47 ± 0.18	88.20 ± 0.40^#^	181.50 ± 0.76	0.28 ± 0.01	−45.00 ± 1.32^#^	3.39 ± 0.01	1.26 ± 0.04
(L3)							
0	99.78 ± 0.28	–	200.60 ± 2.27	0.28 ± 0.11	−41.20 ± 0.44	3.97 ± 0.15	4.23 ± 0.06
1	99.93 ± 1.23	–	192.30 ± 2.35	0.34 ± 0.01	−40.70 ± 0.52	3.68 ± 0.03	4.21 ± 0.05
2	99.10 ± 0.36	–	201.60 ± 2.16	0.25 ± 0.01	−41.00 ± 2.00	3.63 ± 0.02	4.28 ± 0.08
3	99.03 ± 0.30	−	205.90 ± 3.56	0.25 ± 0.02	−39.50 ± 0.72	3.62 ± 0.01	4.30 ± 0.05
**(B)** Ambient conditions (25 ± 2 °C/ 60 ± 5% RH; relative humidity)							
Time	% Drug Content	% EE	PS (nm)	PDI	ZP (mV)	pH	Viscosity (CP)
(F3)							
0	100.20 ± 0.18	90.57 ± 0.98	174.70 ± 7.91	0.33 ± 0.05	−52.30 ± 0.40	3.46 ± 0.01	1.20 ± 0.01
1	99.97 ± 0.25	89.00 ± 0.50	184.80 ± 2.70	0.32 ± 0.01	−44.50 ± 1.95*	3.40 ± 0.01	1.22 ± 0.02
2	99.07 ± 0.40	88.20 ± 0.72*	192.30 ± 1.01*	0.34 ± 0.01	−44.00 ± 2.41*	3.37 ± 0.02	1.21 ± 0.01
3	98.80 ± 0.83*	86.40 ± 0.56*	213.70 ± 3.46*	0.41 ± 0.01*	−42.70 ± 2.52*	3.35 ± 0.01	1.20 ± 0.01
(L3)							
0	99.78 ± 0.28	–	200.60 ± 2.27	0.28 ± 0.11	−41.20 ± 0.44	3.97 ± 0.15	4.23 ± 0.06
1	100.70 ± 1.18	–	207.10 ± 2.36	0.26 ± 0.05	−41.50 ± 0.63	3.70 ± 0.01	4.25 ± 0.04
2	98.80 ± 0.89	–	229.90 ± 2.52*	0.35 ± 0.05	−40.70 ± 1.47	3.63 ± 0.01	4.22 ± 0.08
3	101.20 ± 3.52	–	253.00 ± 2.96*	0.13 ± 0.01	−38.00 ± 0.20*	3.61 ± 0.03	4.27 ± 0.03

* Data are represented as mean ± S.D (*n* = 3). The statistical analysis was performed at *p* < 0.05. # and * indicate significance differences. # vs. the corresponding initial zero-time measurements at the refrigerator temperature. * vs. the corresponding initial zero-time measurements at the ambient conditions. F3: the optimized nanocapsules (NCs), L3: hydroxypropyl chitosan (HPCH) nail lacquer of the optimized NCs. EE%: entrapment efficiency%, PS: particle size, PDI: polydispersity index, ZP: zeta potential..

At ambient conditions, the optimized NCs (F3) exhibited a lower stability than that observed at refrigerator temperature during the three months as reflected by the significantly (*p* < 0.05) changed PS, and EE% during the second and the third months realtive to the intially recorded average values (Table 3B). As well, a significant (*p* < 0.05) lowering in ZP starting from the first month was observed when compared to the intial values. The possible PLGA degradation on storage and the lowered ZP may account for this instability. On the other hand, the nail lacquer (L3) stored at the ambient conditions was more stable than the dispersion as indicated by the statistically insignificant changes of the majority of the examined parameters except the negative ZP which showed a significant (*p* < 0.05) decrease in the third month associated with a significant (*p* < 0.05) increase in PS during the second and third months (Table 3B). The signifcantly (*p* < 0.05) higher viscosity of the lacquer relative to the selected NCs dispersion may still have hindered NCs aggregation.

### 3.4. Nail hydration and in vitro nail plate absorption and desorption

The nail hydration was expressed as a weight gain of the nail clippings following immersion in the tested samples. The average weight gain values were 3.43 ± 0.10, 14.75 ± 1.14 and 3.82 ± 0.16 mg for nail clippings immersed in water (group 1 as a control), nail lacquer of the optimized NCs (group 2), and 1% w/v CIX solution in 1% v/v tween 20 (group 3), respectively. The lacquer of the optimized NCs caused a significant (*p* < 0.05) weight gain in the nail clippings when compred to the water and the drug solution. On the other hand, the weight gain following the immersion in the drug solution was insignificantly different from that produced by water. In agreement, a higher hydration enhancement factor (HE_24_) of the nail lacquer of the optimized NCs (4.30) was recorded when compared to that of CIX (1% w/v) solution (1.11). These results can be attributed to the presence of highly water-soluble film former (HPCH) which is characterized by its high plasticity and affinity toward the keratinized structures such as the nails (Monti et al., 2005). The high hydration tendency is advantageous since the nail swells and behaves like a hydrogel with a network of aqueous pores through which drugs can permeate (Chouhan & Saini, 2012). In most cases of onychomycosis, the fungal infections deeply exist, and hence the drug must reach the underlying tissues of the nails in sufficient concentrations.

Enhanced partitioning of CIX from its lacquer into the nail clippings was proven by the significantly (*p* < 0.05) greater amounts of CIX uptaken (43.21 ± 1.67 µg/mg nail) in comparison with the drug solution (17.70 ± 0.93 µg/mg nail). In accordance, the nail uptake enhancement factor (EF_nail_) of the lacquer relative to the drug solution was 2.60. These results clarified the significantly higher affinity of HPCH nail lacquer to the nail clippings when compared to the drug solution. This superiority may be explained by the prolonged drug release as discussed above as well as the nanometric size and the expected increased permeation of the negatively charged NCs through the nail plate due to the high repulsion forces between them and the negative nail keratin fibers (Elsherif et al., 2017). In addition, HPCH can bind to the nail keratin fibers through its free hydroxypropyl groups by hydrogen bonding (Monti et al., 2005). This interaction can result in an intimate contact with the nail improving the drug permeation in significant amounts enabling effective treatment of onychomycosis (Bseiso et al., 2016).

### 3.5. Microbiological efficacy assessment

#### 3.5.1. Minimum inhibitory concentration (MIC) determination

**Figure 5.**
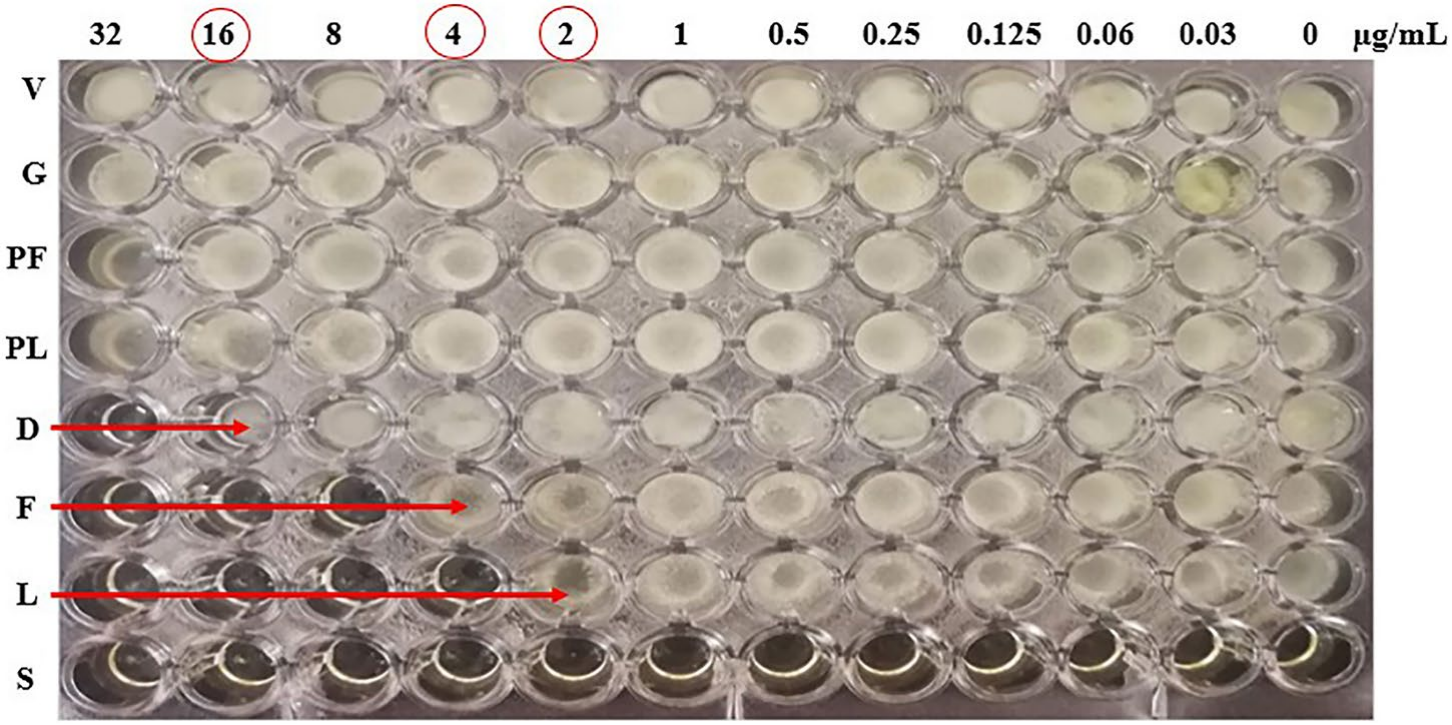
MIC determination by broth microdilution method using the 96-flat-bottomed well microplates. V, solvent control; G, growth control; PF, plain formula (F3); PL, plain lacquer (L3); D, drug DMSO solution; F, medicated NCs (F3); L, medicated lacquer (L3); S, sterility control.

Figure 5 illustrates the results of the micro-dilution test to compare between MIC values of CIX-DMSO solution, the optimized NCs and the tested nail lacquer against *Trichophyton rubrum.* The inspection of fungal growth in the microplates revealed that NCs provided a four-fold decrease in MIC of CIX (4 µg/mL) relative to those recorded for the drug solution in DMSO (16 µg/mL). The possible improved drug diffusion through the fungal membranes due to the prolonged release and the oil content of the negatively charged nanosized capsules as well as PLGA adsorption at the fungal surface and the expected drug deposition at high concentrations on the fungal surface may all account for the greater microbiological efficacy of NCs than CIX solution in DMSO (Lboutounne et al., 2002, Padmavathy & Vijayaraghavan, 2008, Motedayen et al., 2018). More potentiated microbiological efficacy of CIX was achieved by incorporation of these NCs into HPCH nail lacquer that showed an eight-fold decrease in MIC (2 µg/mL) relative to the drug solution. This predominant efficacy of the lacquer over both CIX solution and its optimized NCs may still be explained by its augmented adherence to the fungal cells and the targeted CIX delivery due to HPCH content in the lacquer that could promote the microbiological efficacy (Dillen et al., 2008, Mohammadi et al., 2011). Moreover, HPCH may exert antifungal activity due to its interference with the fungal growth directly and its activation of some defense mechanisms against the fungi, including the accumulation of chitinases, synthesis of proteinase inhibitors and induction of callous synthesis (Peng et al., 2005).

#### 3.5.2. Inhibition zone determination

**Figure 6.**
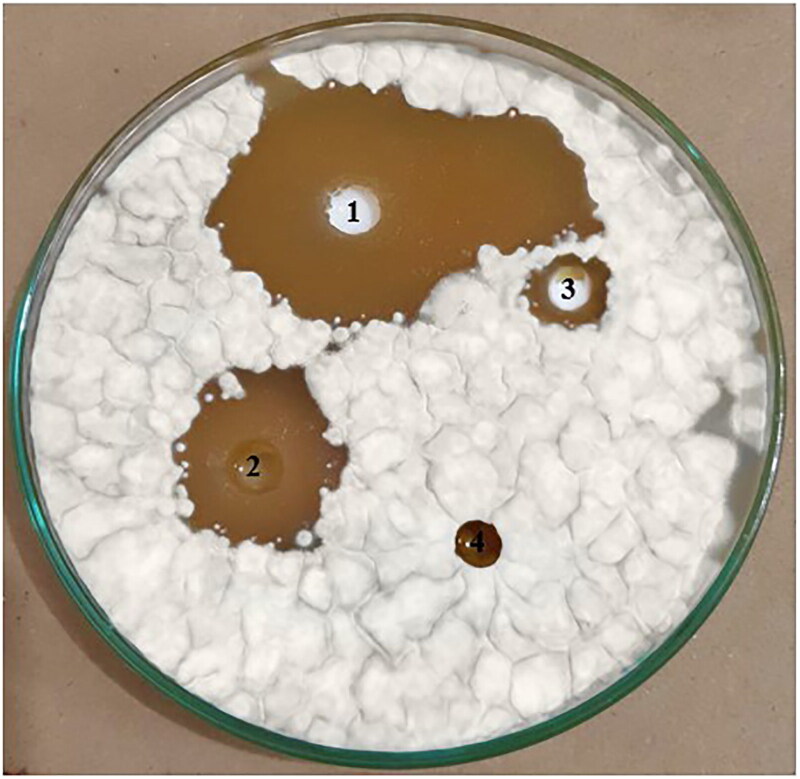
Inhibition zones of CIX nail lacquer of the optimized NCs (1) in comparison with the drug solution (2) plain lacquer (3), and saline (4) against *Trichophyton rubrum* using agar diffusion technique.

The results of *in vitro* antifungal activity through evaluation of inhibition zones of the tested samples are illustrated in (Figure 6). Interestingly, the plain lacquer exhibited an antifungal activity against *Trichophyton rubrum* as suggested by an inhibition zone of a diameter equal to 15.80 ± 0.14 mm. This may be still explained by the antifungal activity of HPCH (Peng et al., 2005). A significantly larger (*p* < 0.05) inhibition zone with a mean diameter of 62.60 ± 13.32 mm was obtained in case of the medicated nail lacquer (L3) when compared to both the drug solution and the plain lacquer. This was followed by that of the drug solution in tween 20 that possessed an average diameter of 33.90 ± 1.11 mm which was found to be statistically greater than that recorded for the plain lacquer. The prolonged release and the expected improved drug diffusion through the fungal membranes due to the oil content of the negatively charged nanosized capsules as well as PLGA adsorption at the fungal surface and the subsequent drug accumulation on the fungal surface may all together with the reported antifungal activity of HPCH explain the greater antifungal activity of NCs nail lacquer than that recorded for the drug solution (Lboutounne et al., 2002, Peng et al., 2005, Padmavathy & Vijayaraghavan, 2008, Motedayen et al., 2018).

#### 3.5.3. Ex vivo evaluation

**Figure 7.**
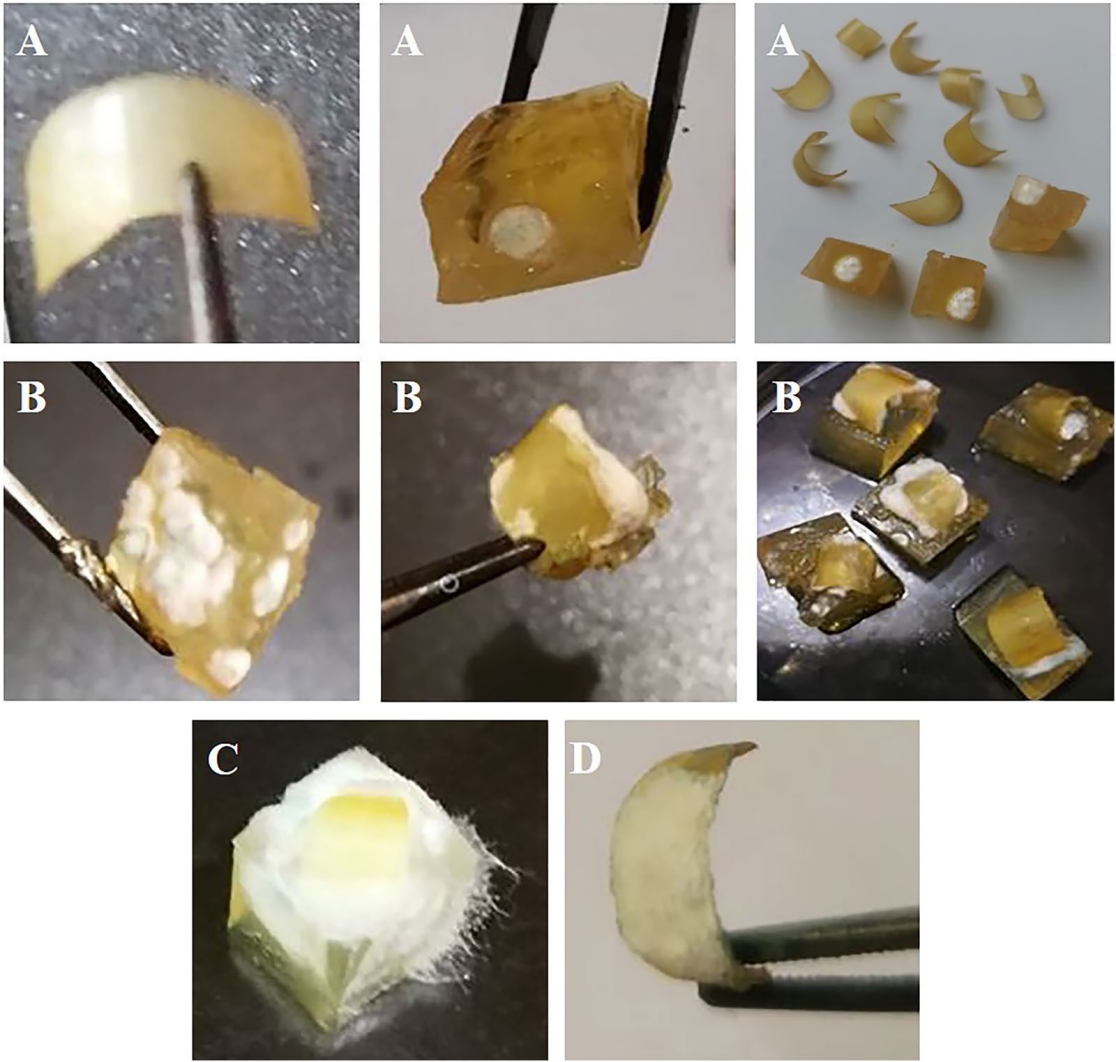
*Ex vivo* onychomycosis model caused by *Trichophyton rubrum* (*n* = 9) showing nail clippings treated with A) CIX nail lacquer of the optimized NCs (L3), B) CIX solution, C) plain lacquer, and D) saline group.

The results of *ex vivo* evaluation of the therapeutic efficacy of the tested samples are shown in (Figure 7). All nail fragments treated with the medicated nail lacquer showed no growth at the nail plate while the fungal growth was restricted to the agar blocks. In contrary, the drug solution exhibited little fungal growth on the nail plates of 7 fragments and moderate growth on the underneath agar blocks. Regarding both plain nail lacquer and saline solution, heavy fungal colonies appeared on the nail fragments and completely covered the agar blocks. The superiority of HPCH nail lacquer of CIX-NCs over its solution may be explained by the ability of PLGA-NCs to maintain CIX soluble and release it in a sustained manner as well as the improved nail permeability by relaxing keratin fibers and pores formation (Gunt and Kasting, 2007, Flores et al., 2017). In agreement, the superior antifungal activity of the nanostructured systems compared to the free drug has been reported in different studies (Flores et al., 2017).

## 4. Conclusions

The optimized NCs of CIX showed a relatively small nanometric diameter as well as high EE% and negative ZP. A prolonged drug release from the optimized NCs and their lacquer was recorded. HPCH nail lacquer of these NCs exhibited a greater storage stability than the optimized NCs. The efficacy against *Trichophyton rubrum* as the causative agent of onychomycosis can be arranged at the following order: the lacquer of the optimized NCs > the optimized NCs dispersion > CIX solution. In conclusion, HPCH nail lacquer of PLGA-NCs can be represented as a promising delivery system of CIX for effective treatment of onychomycosis with a reduced drug dose that can improve the patient compliance and allow cost-effectiveness encouraging further clinical trials.
